# Resveratrol and Its Derivatives in Inflammatory Skin Disorders—Atopic Dermatitis and Psoriasis: A Review

**DOI:** 10.3390/antiox12111954

**Published:** 2023-11-02

**Authors:** Monika Marko, Rafał Pawliczak

**Affiliations:** Department of Immunopathology, Faculty of Medicine, Division of Biomedical Science, Medical University of Lodz, 7/9 Zeligowskiego St., 90-752 Lodz, Poland

**Keywords:** resveratrol, derivatives, inflammatory skin disorders, atopic dermatitis, psoriasis

## Abstract

Atopic dermatitis (AD) and psoriasis are inflammatory skin diseases whose prevalence has increased worldwide in recent decades. These disorders contribute to patients’ decreased quality of life (QoL) and constitute a socioeconomic burden. New therapeutic options for AD and psoriasis based on natural compounds are being investigated. These include resveratrol (3,5,40-trihydroxystilbene) and its derivatives, which are produced by many plant species, including grapevines. Resveratrol has gained interest since the term “French Paradox”, which refers to improved cardiovascular outcomes despite a high-fat diet in the French population, was introduced. Resveratrol and its derivatives have demonstrated various health benefits. In addition to anti-cancer, anti-aging, and antibacterial effects, there are also anti-inflammatory and antioxidant effects that can affect the molecular pathways of inflammatory skin disorders. A comprehensive understanding of these mechanisms may help develop new therapies. Numerous in vivo and in vitro studies have been conducted on the therapeutic properties of natural compounds. However, regarding resveratrol and its derivatives in treating AD and psoriasis, there are still many unexplained mechanisms and a need for clinical trials. Considering this, in this review, we discuss and summarize the most critical research on resveratrol and its derivatives in animal and cell models mimicking AD and psoriasis.

## 1. Introduction

Inflammatory skin diseases such as psoriasis and atopic dermatitis (AD) significantly reduce patients’ quality of life (QoL) and impose a socioeconomic burden [1,2,3,4,5,6]. Over the past several years, there has been a worldwide increase in AD prevalence, especially in developed countries [7,8,9,10,11]. Although the study of the epidemiology of AD is complex and encounters many challenges, the understanding of the incidence of AD has evolved over the past few decades, with emerging trends and new insights about the burden of the disease [12]. It is currently accepted that, depending on the population, AD affects 11–30% of children and 2–10% of adults. In contrast, the prevalence of psoriasis is approximately 1–3% in both children and adults. However, it is a rare skin disorder before the age of 9 [1].

Clinical changes in AD, such as oozing, erythema, and edema, are classified as “acute” [13]. Dyspigmentation, skin dryness, and lichenification are classified as “chronic” changes. The chronic and recurrent nature of the disease should be considered, resulting in the possibility of the coexistence of both types of lesions (“chronic” and “acute”) during exacerbations. AD is characterized by itching and scratching cycles, leading to secondary abrasions. The AD diagnosis is primarily based on the assessment and distribution of lesions and the clinical course of the disease [7,13].

Psoriasis is divided into clinical phenotypes, but the most common and easiest to recognize is chronic plaque or psoriasis vulgaris [5]. Morphologically, psoriasis is characterized by salmon-pink plaques that are well demarcated. The characteristic feature is the anatomical symmetry of the plaques and their color—silvery scales on white skin and gray plaques on black skin [5,14]. Diagnosing psoriasis, as in AD, is based on assessing the morphology and distribution of skin lesions. It is crucial to establish a family history of psoriasis, potential triggers, and musculoskeletal symptoms to diagnose psoriatic arthritis [5]. Despite their important clinical and mechanistic distinctions, AD and psoriasis have a common denominator—red, scaly, and itchy skin lesions. Additionally, the two disorders share disease mechanisms [1,15], such as immune-mediated inflammation and abnormal keratinocyte differentiation [16].

Treating mild-to-moderate AD forms involves topical treatment [17]. This therapeutic line includes emollients [18,19,20,21], topical glucocorticoids (tGCs), and topical calcineurin inhibitors (TCIs) [22,23,24,25]. Topical GCs (tGCs) are highly effective in reducing inflammation and itching. However, tGCs can disturb the skin barrier. In addition, the occurrence of side effects associated with the frequent and long-term use of tGCs and “steroid phobia” cause patients’ reluctance to use them [26].

Treatment strategies for plaque psoriasis should be based on psoriasis severity, location, and multimorbidity [5]. In mild psoriasis, topical agents, including topical corticosteroids, vitamin D analogs, calcineurin inhibitors, and keratolytics, are the mainstay of treatment. Biologics are recommended as a first-line option in moderate-to-severe plaque psoriasis due to their effectiveness and acceptable safety profiles [27,28,29]. Oral treatments include methotrexate, acitretin, cyclosporine, and apremilast [30]. Narrowband ultraviolet B (UVB) phototherapy is also often used [31]. Therapeutic options for psoriasis were revolutionized by the discovery of systemic inflammation’s contribution to the disease’s severity and progression. There are also reports that the strategies used to treat psoriasis should be adopted for the treatment of moderate to severe AD [1].

Although AD and psoriasis have many common features, and their hallmark is inflammation, their immunopathogenesis differs. In AD, the Th2 type of immune response dominates and is characterized by the participation of the cytokines interleukin 4 (IL-4), interleukin 5 (IL-5), interleukin 13 (IL-13), interleukin 33 (IL-33), and thymic stromal lymphopoietin (TSLP) [15]. These proinflammatory cytokines contribute to the impairment of the epidermal barrier typical of AD, the weakening of innate immunity, and the migration of eosinophils. However, the dominant immune response in psoriasis is T helper 17 (Th17), whose characteristic cytokines are interleukin 17A (IL-17A), interleukin 17F (IL-17F), interleukin 21 (IL-21), interleukin 22 (IL-22), interleukin 29 (IL-29), interleukin 8 (CXCL-8), and tumor necrosis factor α (TNF-α). The participation of these cytokines contributes to neutrophil migration, the induction of innate immunity, and excessive epithelial metabolism. In psoriasis, the Th17 response is also reflected by parakeratosis, dilated capillaries, and micro-abscesses [15,32].

Understanding the immunopathogenesis of AD and psoriasis is crucial because currently available therapies specifically target proinflammatory mediators [15]. The participation of a variety of inflammatory mediators in AD and psoriasis immunopathogenesis causes skin cells to be in a chronic inflammatory state. AD and psoriasis are also considered systemic chronic inflammatory diseases [33].

It is worth emphasizing that it was believed that inflammation occurs at the sites of skin lesions associated with psoriasis and atopic dermatitis. However, the severity of these diseases has also been shown to be influenced by the activation of immune mediators in the circulatory system. This is confirmed by the occurrence of comorbidities with atopic dermatitis (“atopic march”) and psoriasis (psoriatic arthritis) of a non-cutaneous nature [1].

Many studies are currently focused on the search for new therapeutic options for AD and psoriasis based on natural compounds such as resveratrol (3,5,40-trihydroxystilbene) and its derivatives. Considering this trend, this review will discuss the most essential research on resveratrol and its derivatives in treating established skin diseases such as AD and psoriasis.

Recently, reviews on the importance of using resveratrol have raised many issues. Among others, Oliveira et al. [34] discuss the potential of resveratrol in inhibiting inflammatory diseases. They describe evidence for the antioxidant and immunomodulatory effects of resveratrol on type 1 diabetes (T1DM), inflammatory bowel diseases (IBDs), psoriasis, rheumatoid arthritis (RA), amyotrophic lateral sclerosis (ALS), and systemic lupus erythematosus (SLE). It is worth emphasizing that the importance of encapsulation in delivery systems to increase resveratrol bioavailability was also discussed.

Also, the importance of resveratrol as an active ingredient for cosmetic and dermatological applications has been reviewed [35]. The influence of resveratrol on the physiological processes taking place in the skin is discussed. Ratz-Łyko et al. [35] reviewed the importance of resveratrol bioavailability and metabolism in the skin. Additionally, its safety was analyzed. Attention was also paid to its anti-aging, skin-whitening, and anti-acne properties. This review also referred to the antioxidant properties of resveratrol. However, the authors of this review primarily addressed the in vitro properties.

Publications on using micro- and nanocarriers for resveratrol delivery into and across the skin for treating different skin diseases deserve special attention [36]. Szulc-Musioł et al. [36] also discussed resveratrol’s role in treating skin diseases. In this case, the authors emphasized the importance of technologies that enhance the transdermal delivery of resveratrol. The authors concluded that this approach might be helpful in solving the problem with the use of resveratrol, which is its low solubility in water and low stability. However, the authors of this review, like Ratz-Łyko et al. [35], referred mainly to in vitro studies, emphasizing the lack of access to clinical trials.

Promising prospects related to using resveratrol and its derivatives in skin diseases were also discussed in the review by Lin et al. [37]. In this case, reference was made to both in vitro and in vivo studies based on cellular and animal models. However, the issue of the lack of clinical trials and the limitations of resveratrol related to its low bioavailability was also raised. A way to overcome these limitations through the topical application of resveratrol and nanocarriers was also addressed. Also, an extensive review of the literature on stilbenoids, including resveratrol, and their anti-inflammatory effects was performed by Dvorakova et al. [38]. The authors also referred to in vitro and in vivo studies in this review. The advantages of this study are its comprehensive discussion of the metabolism of stilbenoids and its attempt to link this knowledge with the impact on human health.

In conclusion, this manuscript complements and summarizes the available information in light of available literature reviews regarding resveratrol. Additionally, it provides an update because it discusses the latest research results from recent years. It is also distinguished because it focuses on two specific diseases—atopic dermatitis and psoriasis—intending to help the reader systematize knowledge. Additionally, the discussed studies include animal studies and in vitro studies.

## 2. Resveratrol and Its Derivatives

Resveratrol was first isolated from the roots of *Veratrum grandiflorum* in 1940 [39]. It was initially known as a phytoalexin, providing defense against insect and pathogen attacks until 1992, when reports emerged showing the cardioprotective effects of red wine [40]. This discovery was related to introducing the term “French paradox”, which refers to improved cardiovascular outcomes despite a high-fat diet in the French population [41]. An explanation for this discrepancy was proposed by Renaud and de Lorgeril [42], who concluded that it was related to the moderate consumption of wine in France (almost 57% of the total consumption of alcoholic beverages). Additionally, they suggested that decreased platelet aggregation may be a significant factor in coronary heart disease [42]. Accordingly, compounds in which red wine is particularly abundant have been identified. Red wine has been proven to be the richest source of resveratrol from the skins, seeds, and stems of the grapes used [41,43]. In addition to grapevines, resveratrol has been identified as a naturally produced phenol and phytoalexin in 72 plant species, including pine trees and legumes. Peanuts, soybeans, and pomegranates are also rich in resveratrol [41]. Resveratrol is classified as a stilbenoid, and its chemical structure is characterized by two phenols connected by an ethylene bridge. There are two isomers: cis- and trans-resveratrol. Trans-resveratrol occurs in plants and is more bioactive than the cis structure. Cis-resveratrol is formed as a result of the isomerization of the trans form and as a result of the breakdown of resveratrol oligomers, which takes place under ultraviolet (UV) radiation during the grape skin fermentation process [37].

Resveratrol derivatives, whose source is also seen in natural resources, have been identified [37,44]. These derivatives were divided into four groups, with stilbene structures substituting moieties in the chemical structure. Resveratrol derivatives include hydroxyl compounds, methoxylated compounds, oligomers, and glycosides [44].

The action of resveratrol and its derivatives covers a broad spectrum of functions. These include antioxidant activities to modulate cellular function and anti-cancer, anti-aging, anti-inflammatory, and antibacterial effects [37,45]. The therapeutic effect of plants containing stilbenoids was already known in folk medicine, where they were used for hepatitis, abdominal pain, arthritis, urinary tract infections, fungal diseases, and inflammatory skin lesions [38,46,47,48].

A limitation of resveratrol is its low bioavailability [45,49]. Resveratrol has also been shown to have a low solubility in water (less than 0.05 mg/mL) [49,50]. These features may result in the insufficient bioavailability of orally administered resveratrol to permit high enough drug concentrations for systemic therapy [51]. Therefore, it can be concluded that these features make it unattractive to pharmaceutical companies despite its beneficial effects on many disorders [31]. An alternative approach to overcoming physicochemical and pharmacokinetic limitations is to use the knowledge of nanotechnology. Thanks to this, it is possible to increase the application possibilities of resveratrol and its derivatives [52]. This approach is based on using nanotechnology-based carriers to encapsulate bioactive natural products such as resveratrol. This strategy involves encapsulating resveratrol in a nanodevice [53]. This method protects against degradation due to contact with the biological environment and facilitates the controlled delivery of bioactive molecules [52,53].

Additionally, nanotechnology helps improve absorption, bioavailability, retention time, and intracellular penetration. Therefore, nanotechnology is a potential strategy to circumvent resveratrol’s physicochemical and pharmacokinetic limitations, which include poor solubility, photosensitivity, and rapid metabolism, which strongly reduce its bioavailability and bioactivity. Nanotechnology achievements such as nanosuspensions, polymeric nanoparticles, nanocapsules, nanofibrous scaffolds, nanoemulsions, nanoliposomes, solid lipid nanoparticles, protein-based nanoparticles, and cyclodextrin allow the attainment of significant therapeutic plasmatic concentrations. At the same time, the toxicological profile can be controlled [52]. Such nanocarriers can reduce drug toxicity and protect unstable molecules from degradation. Additionally, these carriers can control the release of the encapsulated molecule [38]. The kinetics of the release of active substances, their biological activity, and their ability to penetrate biological barriers are influenced by the nanometer size of the carriers [54].

The encapsulation of natural compounds such as resveratrol and its derivatives in specific vehicle formulations (nanocarriers) may also be designed for particular types of administration (oral or topical) and targeted toward specific types of tissue [38]. Interestingly, findings were reported by Nguyen et al. [55] in a study on long-acting resveratrol/metformin nanotherapeutics that may be applicable to the treatment of macular degeneration. For this study, a potent nanotherapeutic named R@PCL-T/M NPs was designed. The development of R@PCL-T/M NPs was based on the aminolysis of resveratrol-encapsulated polycaprolactone nanoparticles (R@PCL NPs). This stage was followed by the formation of amide linkages with carboxyl-terminated transacting activators of transcription cell-penetrating peptide (T) and metformin (M). Then, its potential use in the intravitreal treatment of wet age-related macular degeneration (AMD) was assessed. Experiments were performed in vitro and in vivo levels in a rat model. The in vitro results indicated that nanotherapeutics have antioxidant, anti-inflammatory, and anti-angiogenic effects in biological environments. In contrast, experiments on an AMD rat model showed that a single-dose intravitreal injection of R@PCL-T/M NPs could alleviate disease progression for 56 days.

Using nanotechnology to improve resveratrol delivery may represent a novel approach. However, further in vivo studies on the toxicity of nanotechnology-based carriers need to be conducted. In this way, it will be possible to conduct more extensive, randomized clinical trials and evaluate the potential of nanotechnology in the delivery of resveratrol [52].

According to Shaito et al. [56], although numerous scientific reports have provided extensive knowledge and data on the beneficial effects of resveratrol on many diseases, relatively few reports analyze its toxicity. Differences in the methodology of the conducted studies, such as the characteristics of the patients enrolled, the size of the dose, and the time of administration of resveratrol, also raise doubts. There are currently no studies examining the adverse effects and the possibility of interactions between resveratrol and other therapies in humans.

## 3. Resveratrol and Its Derivatives in Inflammatory Diseases

Resveratrol and other natural stilbenoids, such as piceatannol, pterostilbene, and gnethol, are compounds with proven anti-inflammatory effects both in vitro and in vivo [38]. These non-nitrogen polyphenols with an acidic and amphiphilic character target COX-2, 5-lipoxygenase (5-LOX), and protein kinase B (PKB/Akt) [38,57,58,59]. Zhou et al. [60] assessed the anti-inflammatory effects of resveratrol on a rabbit model of acute pharyngitis. Also, Wang et al. [61], in an animal model of mice in which ear edema was induced with xylene, showed that resveratrol has strong analgesic and anti-inflammatory effects. The anti-inflammatory effect of resveratrol has also been demonstrated in studies on neurological disorders [62] and bowel disease [63,64].

The use of resveratrol in treating inflammatory diseases has been evaluated in many studies, including those involving rodents. The results of these studies contributed to broadening the knowledge about the pharmacology of resveratrol and its potential use [55,65].

Resveratrol has been shown to reduce the levels of proinflammatory mediators such as interleukin 1β (IL-1β), interleukin 6 (IL-6), TNF-α, and TGF-β1 in a rat model of Crohn’s disease [66]. Additionally, in a murine model of hyper-acute Th1-type ileitis, following peroral infection with Toxoplasma gondii, resveratrol, curcumin, and simvastatin decreased the expression of IL-23p19, IFN-γ, TNF-α, IL-6, and MCP-1 [67].

Moreover, in mouse models of chronic colonic inflammation, resveratrol reduced the rise in TNF-α and IL-1β and increased the anti-inflammatory cytokine IL-10. In addition, resveratrol reduced the expression of prostaglandin E synthase-1 (PGES-1), cyclooxygenase-2 (COX-2), and inducible nitric oxide synthase (iNOS) protein expression [66,67,68].

Significantly, in an acute pancreatitis (SAP) rat model treated with resveratrol when proinflammatory mediators were downregulated, there was a concomitant upregulation of inflammation-reducing biomarkers, including antioxidant proteins such as superoxide dismutase (SOD), with the downregulation of malondialdehyde (MDA) [69]. In another SAP rat model study, reductions in nitric oxide (NO) and endogenous angiotensin II and endothelin were observed after resveratrol administration [70].

Also, cell studies have assessed the effect of resveratrol on the production of proinflammatory cytokines [38]. Among others, Djoko et al. [71] conducted a study on macrophages and deduced that stilbenoids effectively inhibit prostaglandin E2 (PGE2)- or NO-mediated inflammation. The inhibition of NF-κB or CCAAT/enhancer-binding protein δ (C/EBPδ) has also been shown to mediate the expression of inflammatory genes. In a further study, Chung et al. [72] showed that resveratrol can downregulate interferon-γ-induced inflammatory genes in macrophage cell lines. Additionally, Bi et al. [73] revealed that resveratrol may act as an inhibitor of the production of NO and TNF-α by lipopolysaccharide-activated microglia in murine cell lines, whereas Shakibaei et al. [74], in their human articular chondrocyte study, demonstrated that resveratrol suppresses IL-1β-induced inflammatory signaling and apoptosis. What is more, Oh et al. [75] described resveratrol as having anti-inflammatory effects by inhibiting interleukin-8 (IL-8) production in a lipopolysaccharide (LPS)-induced human monocyte cell line. Kang et al. [76] demonstrated the effects of resveratrol on the expression of proinflammatory cytokines in the human mast cell line. Gonzales et al. [77] conducted a study using adipocyte cell lines incubated with resveratrol and curcumin. This study showed that the tested compounds can inhibit TNFα-activated NF-κB signaling and, as a result, significantly reduce the expression of proinflammatory cytokines. Also, piceatannol showed anti-inflammatory activity in mast-cell-mediated allergic inflammation. It was observed that piceatannol inhibited histamine release from rat peritoneal mast cells and reduced immunoglobulin E (IgE)-mediated reactions [78].

Considering the issue of resveratrol’s involvement in the mechanism of inflammation, its antioxidant effect and ability to scavenge free radicals should be emphasized [57,79]. Inflammation is associated with producing massive amounts of reactive oxygen species (ROS), which are critical suppressors of inflammation, as they initiate neutrophil apoptosis. Moreover, ROS mediate toxic oxidative reactions, damaging proteins, nucleic acids, and lipids, especially during prolonged inflammatory and oxidative reactions [38].

It has been proven that there is a connection between oxidative stress and the proper functioning of the skin [80]. It is known that the skin has a barrier function and is constantly exposed to environmental factors. Therefore, it is a source of free radicals [81]. On the other hand, an excessive increase in the production of oxygen and nitrogen free radicals or a decrease in the ability of antioxidant systems to eliminate them leads to a phenomenon called oxidative stress [82]. An increase in the level of free radicals or a disturbance of their balance is then noticeable. This can lead to changes such as the degradation of cellular proteins, lipid oxidation, cell apoptosis, tissue damage, altered T helper cell (Th cell) response, and interleukin-17 (IL-17) secretion [83,84]. It is also assumed that free radicals may be involved in processes affecting the formation of changes in DNA [83].

Many literature items describe the relationship between oxidative stress and inflammation [85]. It has also been proven that oxidative stress can cause or exacerbate skin inflammation [86]. The anti-inflammatory effect of polyphenols is based on scavenging free radicals and regulating cellular activity in inflammatory cells [85,87]. These natural compounds also modulate the activity of enzymes involved in arachidonic acid metabolism [85]. This is of particular importance in anti-inflammatory mechanisms, the aim of which is to inhibit eicosanoid-producing enzymes, including phospholipase A2 (PLA2), cyclooxygenase (COX), and lipoxygenase (LOX), which leads to a decrease in the concentration of prostanoids and leukotrienes [88]. Polyphenols also contribute to arginine metabolism and modulate the production of other proinflammatory molecules [77]. Dvorakova et al. [38], in a 2017 review, draw attention to research results showing that resveratrol suppresses the production of PGE2 and NO and inhibits COX-2, iNOS proteins, and mRNA.

In vitro and in vivo studies demonstrated that atopic skin is susceptible to oxidative stress [89,90,91]. Studies have shown that oxidative stress and altered antioxidant defense are involved in the pathogenesis of AD. These findings provide new opportunities for novel AD treatment strategies that inhibit oxidative stress [90]. Also, the role of oxidative stress has been demonstrated in the development of inflammation in psoriasis [84,92,93,94]. Although oxidative stress markers have been shown to be elevated in psoriasis and related to disease duration and severity, further research is needed to investigate the usefulness of assessing oxidative stress biomarkers and apply these findings to treating psoriasis [84].

## 4. Resveratrol and Its Derivatives in Atopic Dermatitis and Psoriasis

The skin is the outermost organ and separates the body from the external environment. As a result, it is constantly exposed to external pathogens and chemical, physical, and microbiological factors. To maintain homeostasis and overcome inflammation resulting, among others, from exposure to harmful external factors, immune cells in the skin are activated to orchestrate skin immune responses [95]. On the other hand, inflammation is crucial in the pathogenesis of some skin diseases, such as AD or psoriasis [37,96]. Resveratrol’s therapeutic potential in reducing skin inflammation has been reported in psoriasis studies [34,37]. It has been shown that by activating the sirtuin 1 (SIRT1) mechanism, resveratrol inhibits aquaporin 3 (AQP-3), which regulates cell survival. This, in turn, reduces keratinocyte proliferation, characteristic of psoriasis [97].

Although the low bioavailability and low water solubility of resveratrol have been demonstrated, making it challenging to use in systemic administration [49], it may play a role in topical therapy [37]. The use of resveratrol in topical treatment is promising because it has been found to be easily absorbed through the skin [51]. For a therapeutic agent to be properly delivered to the skin, it must have the appropriate particle size and physicochemical properties [98]. Because the stratum corneum (SC) is resistant to topical medications, the ideal therapeutic agents should be characterized by low molecular weight (<500 g/mol), moderate lipophilicity (partition coefficient log P 1/4 1–3), and adequate solubility in both water and oil, as well as have a low melting point [37,98]. These properties facilitate effective drug delivery through the skin, and resveratrol and its derivatives meet these criteria [37]. In this context, it has been concluded that resveratrol and some of its derivatives are promising candidates for topical application in treating skin diseases. Therefore, there is a premise for analyzing and conducting new research on resveratrol and its derivatives to check their therapeutic potential in topical treatment [37]. In addition, the possible likelihood of skin irritation from topical application of resveratrol is low [99]. According to Lin et al. [37], resveratrol can ameliorate the signs and symptoms of AD and psoriasis. Table 1 summarizes the stage of action of resveratrol and its derivatives in AD and psoriasis.

**Figure 1:**
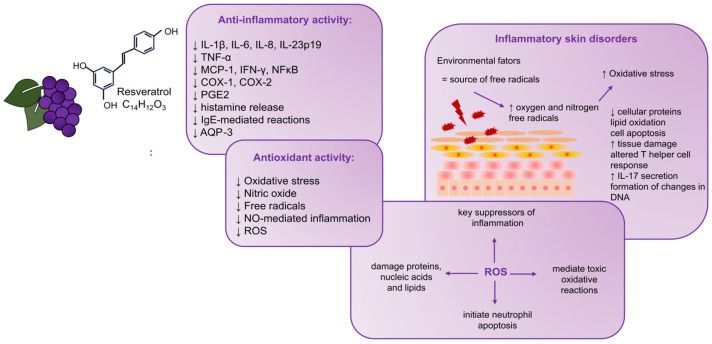
The relationships between resveratrol’s anti-inflammatory and antioxidant effects and its derivatives in developing inflammatory skin disorders. Resveratrol may affect inflammation through its antioxidant activity and scavenging of free radicals. Key inflammation suppressors are ROS because they trigger the apoptosis of neutrophils. There is a connection between oxidative stress and the proper functioning of the skin. The phenomenon of oxidative stress, an increase in the level of free radicals or their imbalance, can lead to the degradation of cellular proteins, lipid oxidation, cell apoptosis, tissue damage, altered T helper cell response, secretion of IL-17, and changes in DNA, which, in turn, can lead to skin disorders. IL-1β: interleukin 1β; IL-6: interleukin 6; IL-8: interleukin 8; IL-23p19: interleukin 23p19; TNF-α: tumor necrosis factor α; MCP-1: monocyte chemoattractant protein 1; IFN-γ: interferon-γ; NFκB: nuclear factor kappa B; COX-1: cyclooxygenase-1; COX-2: cyclooxygenase-2; PGE2: prostaglandin E2; IgE: immunoglobulin E; AQP-3: aquaporin 3; NO: nitric oxide; ROS: reactive oxygen species; IL-17: interleukin-17.

AD and psoriasis are disorders that, in their pathogenesis, show many similarities, ranging from the deregulation of the immune response through genetic determinants to abnormalities in keratinocyte proliferation. As a result, both conditions are characterized by dry, scaly skin. In order to accurately reflect the course of the pathogenesis of these diseases, cellular models are increasingly used [100]. In AD and psoriasis research, in addition to in vitro models, in vivo animal models are used [101,102,103]. Although animal models are widely used for AD and psoriasis research, researchers should be cautious when extrapolating their data to human diseases. This is dictated by the fact that many discrepancies have been noticed between animal models and the course of the disease in humans. Typically, these are differences in inflammatory profiles or the fact that human AD can be divided into several phenotypes and endotypes and distinct types depending on the patients’ age, chronicity, or genetic background, which influences the course of its pathogenesis [101,103]. In the case of psoriasis, it is crucial to be aware that similar hyperproliferative inflammatory phenotypes can result from different manipulations. Therefore, it was concluded that no animal model described so far reflects all aspects of human psoriasis [102]. Figure 1 summarizes the described relationships between resveratrol’s anti-inflammatory and antioxidant effects and its derivatives in developing inflammatory skin disorders.

### Overview of Selected Studies

High-mobility group box 1 protein (HMGB1) was discovered to be a crucial cytokine that mediates the response to infection, injury, and inflammation [104]. The study by Karuppagounder et al. in 2014 [105] was aimed at investigating the ameliorative potential of resveratrol on HMGB1 signaling and skin inflammation in NC/Nga mice. The NC/Nga mice have been developed explicitly for the study of AD and are widely used as models since they exhibit pathological and behavioral features similar to AD [106,107]. Given that AD patients are highly sensitized to house dust mite (HDM) allergens [108,109], in this study [105], an HDM-induced AD mouse model was used. Dermatophagoides farinae body extract (DFE)-cream-treated mice (100 mg/mouse) were divided into two groups, and each received either the vehicle or resveratrol (20 mg/kg/day, orally by gavage). The control was normal, untreated mice. A mouse model was used in which AD was induced by DFE, similar to an earlier study by a Korean team that investigated the properties of ginseng in AD [110]. In the described study [105], dermatitis severity, histopathological changes, serum levels of IFN-γ and interleukin 4 (IL-4), and changes in the protein expression of HMGB1 were evaluated by Western blotting. In addition, inflammatory markers in the skin of AD mice, such as receptors for advanced glycation end products (RAGE), toll-like receptor 4 (TLR4), NFκB, phosphatidylinositide 3-kinase (PI3K), extracellular signal-regulated kinase 1/2 (ERK1/2), COX2, TNFα, IL-1β, and interleukin 2 receptor alpha (IL-2Rα), were assessed. It can be concluded that due to the complexity of the study design and numerous observations, its results significantly contribute to expanding current knowledge. The described study showed that resveratrol inhibited AD-like skin lesion development. Furthermore, the histological analysis provided information that resveratrol inhibited hypertrophy, intracellular edema, mast cells, and the infiltration of inflammatory cells. It should be emphasized that resveratrol treatment downregulated HMGB1, RAGE, phosphorylated nuclear factor-κB (p-NFκB), phosphoinositide 3-kinase (p-PI3K), phosphorylated extracellular signal-regulated kinase (p-ERK1/2), COX-2, TNFα, IL-1β, IL-2Rα, IFNγ, and IL-4. The obtained outcomes shed light on the HMGB1 pathway as a potential therapeutic target in skin inflammation. Whereas resveratrol can modulate HMGB1 protein expression, its administration could benefit AD. Although these findings suggest that resveratrol may be an effective therapeutic agent for AD, this study should be interpreted cautiously, as it is limited to only one resveratrol dose.

The issue of the impact of resveratrol on mechanisms related to AD was also addressed by Sozmen et al. in 2016 [111]. The research team from Turkey evaluated how resveratrol affects epithelial-derived cytokines and epithelial apoptosis in a mouse model reminiscent of AD. In this model, AD was induced by 2,4-dinitrophenylbenzene (DNFB) in BALB/c mice. What is crucial is that this study showed significantly improved epithelial thickness in the resveratrol group compared with the vehicle group. Additionally, the study revealed a lower number of interleukin-25 (IL-25)-, IL-33-, and TSLP-positive cells in the epithelium after resveratrol treatment compared to the vehicle group. Also, in the resveratrol group, the number of caspase-3-positive cells in the epithelium was significantly lower compared to the vehicle group as an indicator of apoptosis. Analyzing the results of this study, resveratrol, through its action on epithelial cytokines and epithelial apoptosis, effectively alleviates histological changes and the first stage of inflammation. Although the study showed that resveratrol may have therapeutic benefits, the authors emphasized that it is too early to draw definitive conclusions.

On the other hand, Bangash et al. in 2023 [112], turned to a compound that is structurally similar to resveratrol called pterostilbene, a naturally occurring antioxidant. Pterostilbene is a phytoalexin found in the grapevine and has similarly profound anti-inflammatory, antioxidant, and anti-cancer properties to resveratrol. It should be noted that pterostilbene is worth attention due to its better bioavailability compared to resveratrol. It is more lipophilic due to the presence of two methoxy groups. This study aimed to evaluate the effect of pterostilbene in a 2,4-dinitrochlorobenzene (DNCB)-induced AD-like mice model. Mice were assigned to four groups: one control group received topical 0.1% tacrolimus, and three received daily topical pterostilbene at 0.2, 0.6, and 1%. The obtained outcomes revealed that pterostilbene treatment reduced the elevated IgE level and blood inflammatory cells in AD-like mice. An improvement in the number of oxidative stress markers in the skin of the diseased mice was also observed. The obtained results can be considered necessary in terms of the relationship between oxidative stress and proper skin function. Moreover, reduced expression levels of IL-4, IL-6, TNF-α, and NF-κB in the skin of diseased mice were demonstrated after pterostilbene treatment. Additionally, a decreased epidermal thickness in pterostilbene-treated groups was observed. Considering the results described in this study, it can be concluded that pterostilbene may alleviate AD symptoms by reducing the inflammatory state, the inflammatory cytokines, and oxidative damage in mice’s skin.

Interesting observations were described by Kwack et al. in 2022 [86]. A study conducted in Korea evaluated the preventative effects of antioxidants against coarse particulate matter <10 μm (PM10) on the serum IgE concentration, mast cell counts, inflammatory cytokines, and keratinocyte differentiation markers. The PM assessed in this study is generated by factories, power plants, cars, and construction activities [113]. Also, natural dust that the wind carries can be a PM source. It has been proven that it can be an environmental stressor, affecting the human body through the synthesis of ROS [86,114]. Oxidative stress can cause disorders of the respiratory and cardiovascular systems and exacerbate external skin aging and skin inflammation [115]. In this study, a DNCB-induced AD-like BALB/c mice model was used. Antioxidants such as dieckol, punicalagin, epigallocatechin gallate (EGCG), Siegesbeckiae Herba extract (SHE), resveratrol, and PM10 were applied and investigated. After using PM10, increases in the serum IgE concentration, mast cell count, inflammatory cytokines, and keratinocyte differentiation markers were observed. However, the presence of antioxidants modulated these changes by inhibiting the upregulation of inflammatory cytokines: (IL)-1β, IL-4, IL-6, interleukin 17 α (IL-17α), IL-25, interleukin 31 (IL-31), and TSLP. Additionally, whereas PM10 downregulated the expression of keratinocyte differentiation markers, including loricrin and filaggrin, antioxidants prevented the downregulation of these markers.

**Figure 2:**
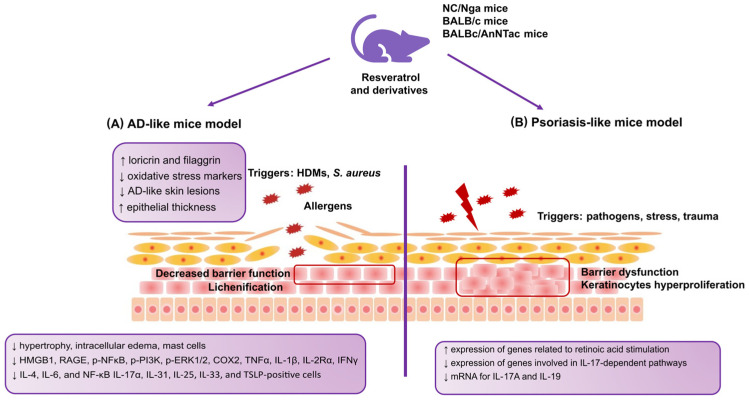
The relationships and molecular pathways of resveratrol and its derivatives reported in studies on AD-like and psoriasis-like cell models. (**A**) Changes observed after using resveratrol and its derivatives in a cellular model mimicking AD; (**B**) changes observed after using resveratrol and its derivatives in a cellular model mimicking psoriasis. AD: atopic dermatitis; HDMs: house dust mites; HMGB1: high-mobility group box 1 protein; RAGE: receptors for advanced glycation end products; pNF-κB: phosphorylated nuclear factor-κB; p-PI3K: phosphoinositide 3-kinase; p-ERK1/2: phosphorylated extracellular signal-regulated kinase; IL-2Rα: interleukin-2 receptor α; IL-4: interleukin 4; IL-17α: interleukin 17 α; IL-31: interleukin 31; IL-25: interleukin 25; IL-33: interleukin 33; TSLP: thymic stromal lymphopoietin; IL-17A: interleukin 17A, IL-19: interleukin 19.

A psoriasis-like model was used by Kjaer et al. in 2015 [116]. Researchers from Denmark reported the results of a study that focused on the ameliorating effect of resveratrol on psoriasis-like skin lesions. Inflammation was induced in BALBc/AnNTac mice by Imiquimod (IMQ). This study showed that resveratrol significantly reduced the severity of psoriasis-like dermatitis in mice after IMQ treatment. A resveratrol-dependent change in the gene expression profile was observed, such as an increase in the expression of genes related to retinoic acid stimulation and a decrease in the expression of genes involved in IL-17-dependent pathways. In addition, mRNA decreased for both interleukin 17A (IL-17A) and interleukin 19 (IL-19), which are crucial in developing psoriasis. These results suggest that resveratrol may be a potential treatment option for psoriasis and should be investigated in further studies to apply the results to humans. Figure 2 summarizes the relationships and molecular pathways of resveratrol and its derivatives reported in studies on the AD-like and psoriasis-like animal models described in this review.

Kang et al. in 2019 [117], used NC/Nga mice in their study on the effects of resveratrol-enriched rice on skin inflammation and pruritus. A team of researchers from Korea decided to test the potential of rice (*Oryza sativa var. japonica*), consumed by Asians in large quantities, in their study. It is also widely used in the cosmetic industry. Rice enriched with resveratrol (RR) was used, specifically a transgenic rice variety showing the synergistic effect of resveratrol and rice. It was shown that RR may have synergistic effects stronger than those of either resveratrol or rice alone, and RR grains may contain as much resveratrol as high-quality red wine. The study investigated the inhibitory effect of RR on AD-like skin lesions in vitro and in vivo. A mouse model induced by DNCB was used, similar to the previously mentioned study [111] that showed that resveratrol effectively reduced inflammation and histological changes by modulating apoptosis and regulating cytokine secretion in the epithelium. The authors of the described study reported interesting results regarding a significant reduction in scratching frequency and AD severity. They also observed improved trans-epidermal water loss (TEWL) and mice’s skin hydration. Additionally, to investigate the optimal effective range of concentrations of RR in human keratinocytes, cells (HaCaT) were treated with normal rice, resveratrol, and RR. After cells were incubated with RR, significant decreases in IL-31 and IgE levels and the suppression of the production of IL-6 in the culture medium of human keratinocytes and a 3D skin model were observed. Taking into account the results of this study, it can be concluded that the topical application of RR can be used as a potential alternative therapy for inflammatory skin diseases such as atopic dermatitis.

**Figure 3:**
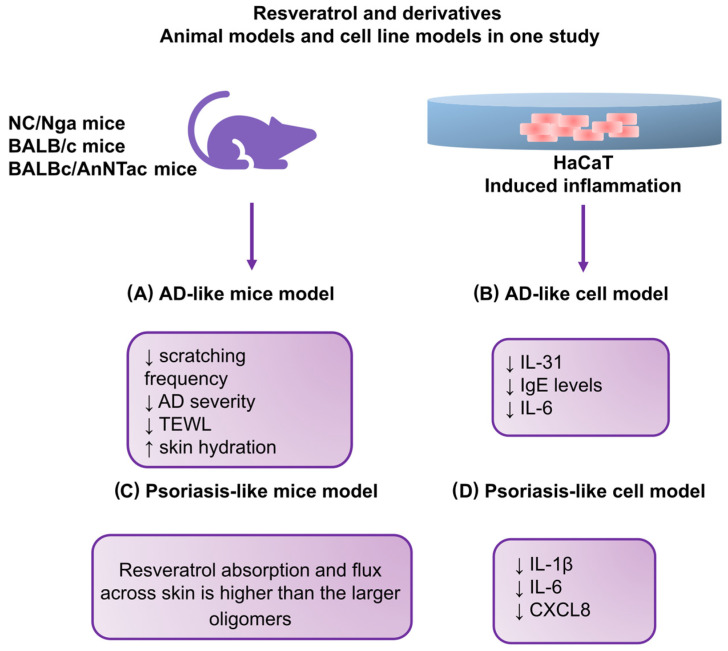
The relationships and molecular pathways of resveratrol and its derivatives reported in studies on AD-like and psoriasis-like animal models. (**A**) Changes observed after using resveratrol and its derivatives in a mouse model mimicking AD; (**B**) changes observed after using resveratrol and its derivatives in a cellular model mimicking AD; (**C**) changes observed after using resveratrol and its derivatives in a mouse model mimicking psoriasis; (**D**) changes observed after using resveratrol and its derivatives in a cellular model mimicking psoriasis. HaCaT: human keratinocyte cell line; TEWL: transepidermal water loss; CXCL8: interleukin 8.

Additionally, Cheng et al. [118] conducted a comprehensive psoriasis study that evaluated the efficacy of the topical administration of resveratrol oligomers. In vivo and in vitro psoriasis-like models were used to explore the effect of the number of resveratrol subunits on skin absorption to establish the structure–permeation relationship (SPR). The SPR provides insight into the effects of physicochemical characteristics on skin absorption and is helpful in the development of topical therapies [119]. HaCaT keratinocytes activated with IMQ were used to assess cytokine/chemokine inhibition. The Franz diffusion cell system was used to evaluate delivery to porcine skin. IMQ was also administered to Balb/c mice to study skin absorption, skin physiology, the expression of proinflammatory mediators, and histopathology after the topical application of resveratrol and oligomers. In vitro experiments in keratinocyte cell lines showed that resveratrol and its derivatives had a similar ability to inhibit the proinflammatory cytokines IL-1β, IL-6, and CXCL8. The study stage aimed at assessing the absorption capacity of the tested compounds in the skin showed that resveratrol achieved the best results. The effect of ε-winiferine was slightly worse. In addition, the study indicated the importance of the particle size and lipophilicity of permeants in skin deposition (the larger the particles, the less skin deposition), which may be necessary for developing topical therapies. The research was extended with the introduction of in silico modeling. In this stage, it was shown that in the skin of mice with impaired epidermal barrier function, the concentration of ε-viniferin was higher than that of resveratrol. It was also observed that permeants that strongly interact with the lipids of the stratum corneum are less transported to the skin. This study revealed that topical ε-viniferin alleviated psoriasiform symptoms and reduced interleukin 23 (IL-23) secretion more effectively than resveratrol. The results of this study shed light on the importance of the dimer structure, which may be a basis for the further design of new antipsoriasis agents. Figure 3 summarizes the relationships and molecular pathways of resveratrol and its derivatives reported in studies on the AD-like and psoriasis-like animal and cellular models described in this review.

Shin et al. (2020) [120], similar to the previously mentioned study by Kwack et al. [86], assessed particulate matter (PM) and its association with inflammation. In contrast to the study by Kwack et al. [86], Shin et al. evaluated only resveratrol and used an in vitro model of normal human epidermal keratinocytes (NHEKs). The authors justified this issue by reporting that PM causes intracellular inflammation in human keratinocytes and is associated with various skin diseases, including AD. Therefore, in this study, an attempt was made to learn the molecular mechanisms underlying the action of resveratrol and its beneficial effect on skin changes caused by exposure to PM. Based on the results obtained, the authors concluded that resveratrol could potentially prevent skin disorders associated with air pollution. The main reasons for this conclusion were that outcomes suggested that resveratrol can inhibit PM-induced aryl hydrocarbon receptor activation. In addition, the authors observed that resveratrol can reduce reactive oxygen species formation in keratinocytes.

The study’s authors also suggested that resveratrol inhibits the expression of proinflammatory mediators such as matrix metalloproteinase 1 (MMP-1), matrix metalloproteinase 9 (MMP-9), and IL-8. A decrease in the expression of COX-2/PGE2 was also observed.

It is worth emphasizing that mast cell lines are also used to study AD. The success of using these cells is confirmed by the study results reported by Moon et al. (2020) [121]. In the described study, the authors evaluated the effect of resveratrol on an AD mast cell model induced with phorbol myristate acetate (PMA) plus calcium ionophore (PMACI). Since TSLP plays a key role, among others, in the pathogenesis of AD, and the effect of resveratrol on the regulation of TSLP has not been clarified, the authors conducted a study in which they undertook to investigate this mechanism in human mast cells (HMC-1). An important observation was that in cells activated with PMACI and incubated with resveratrol, there were decreases in the activation of NFκB, the phosphorylation of NF-kappaB inhibitor alpha (IκBα), and the activation of receptor-interacting protein 2 and caspase-1. Additionally, lower TSLP production and mRNA expression were observed. The results concluded that resveratrol may be helpful in treating diseases such as asthma, allergic rhinitis, and AD by blocking TSLP.

**Figure 4:**
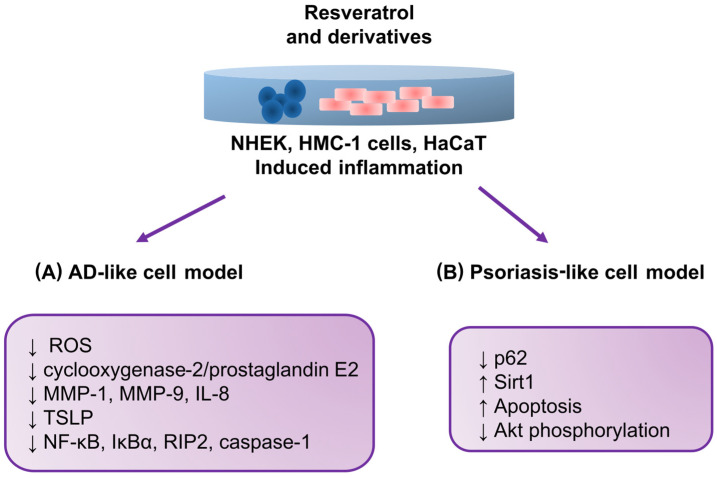
The relationships and molecular pathways of resveratrol and its derivatives reported in studies on AD-like and psoriasis-like cell models. (**A**) Changes observed after using resveratrol and its derivatives in a cellular model mimicking AD; (**B**) changes observed after using resveratrol and its derivatives in a cellular model mimicking psoriasis. NHEKs: normal human epidermal keratinocytes; HMC-1: human mast cell line; MMP-1: matrix metalloproteinase 1; MMP-9: matrix metalloproteinase 9; IκBα: NF-kappaB inhibitor alpha; RIP2: receptor-interacting protein-2 kinase; p62: protein p62; Sirt1: sirtulin 1, Akt: protein kinase B (PKB).

Relevant outcomes were obtained by Lee et al. (2016) [122], who evaluated the effect of resveratrol on cell death in a psoriasis-like HaCaT cell model. This study indicated the potential of resveratrol in treating psoriasis by demonstrating a relationship in the molecular pathway involving a reduction in p62 protein levels. As a result, apoptosis occurred in the examined keratinocytes. The induction of sirtulin 1 (Sirt1) by resveratrol was also observed, resulting in the inhibition of protein kinase B (Akt) phosphorylation. Figure 4 summarizes the relationships and molecular pathways of resveratrol and its derivatives reported in studies on the AD-like and psoriasis-like cell models described in this review.

A summary of the data characterizing the original studies discussed above is presented in Table 2.

## 5. Conclusions and Future Directions

This review covers the most important research on resveratrol and its derivatives’ role in AD and psoriasis. Although there is access to the results of numerous studies in this field, these are primarily studies conducted on cell and animal models. There are still no reports from clinical trials that could finally confirm the potential of resveratrol and its derivatives as a natural compound in treating inflammatory skin diseases. Therefore, it can be concluded that there are still grounds for conducting further research in this area, which will focus on developing therapeutic strategies in humans. Our review also found that some studies reported differences in their methodology. In particular, there were differences in the concentrations of the tested substances, which varied significantly between studies. However, it can be concluded from this review that resveratrol and its derivatives are promising compounds, according to animal and cell studies focusing on aspects related to the course of atopic dermatitis and psoriasis. The results of these studies constitute a rationale for conducting research on AD and psoriasis therapies based on the use of resveratrol and its derivatives. However, their mechanism of action in humans needs to be thoroughly investigated, as the current knowledge is mostly based on results from studies on animals and cell lines. One should also be aware that these models have limitations and do not fully reflect the course and pathogenesis of the disease but only resemble it. Therefore, researchers should interpret with caution the results obtained in studies based on cell models and animal models mimicking AD and psoriasis. It is also essential to remember resveratrol’s low solubility and bioavailability and consider possible options to improve this condition if resveratrol is considered a potential therapeutic agent. Taking into account the promising results of studies on the topical use of resveratrol and its derivatives in mice, it can be concluded that this form of administration may be more effective than systemic supplementation. However, existing research results in this field require confirmation in clinical trials. In addition, it should be emphasized that there is still a lack of research on adverse effects and the possibility of interactions of resveratrol with other therapies in humans. This review also shows that more studies have been reported on resveratrol than on its derivatives. These conclusions suggest that resveratrol and its derivatives still have an unexplored side in treating AD and psoriasis.

## Figures and Tables

**Table 1:** The role of resveratrol and its derivatives in atopic dermatitis and psoriasis. Stages of action [37].

Resveratrol and Its Derivatives—Stages of Action	
Atopic dermatitis	↑ Skin barrier function ↓ Scratching ↓ Edema ↓ Hyperplasia ↓ Immune cell infiltration
Psoriasis	↓ Keratinocyte proliferation ↓ Hyperplasia ↓ Plaque ↓ Neutrophil infiltration

**Table 2:** Characteristics of included studies.

Authors [Reference]	Year	Cell/Animal/ Model Type	Induction Methods	Resveratrol Dosage	Molecular Target and Effect
Karuppagounder et al. [105]	2014	NC/Nga mice; AD-like model	HDM extract (100 mg of DfE cream)	20 mg/kg for 2 weeks	↓ Hypertrophy ↓ Intracellular edema ↓ Mast cells ↓ Infiltration of inflammatory cells ↓ HMGB1 ↓ RAGE ↓ p-NFκB ↓ p-PI3K ↓ p-ERK1/2 ↓ COX2 ↓ TNFα ↓ IL-1β ↓ IL-2Rα ↓ IFNγ ↓ IL-4
Kang et al. [117]	2019	NC/Nga mice; AD-like model	200 μL of 0.4% DNCB solution	RR; 2.5%, 5 mg/200 μL; and DNCB + resveratrol 2.5%, 5 mg/200 μL	↓ Scratching frequency ↓ Dermatitis severity ↓ TEWL ↑ Skin hydration
Kang et al. [117]	2019	HaCaT and a 3D skin model; AD-like model	TNF-α and IFN-γ (each 10 ng/mL)	RR 10 ng/mL	↓ IL-31 and IgE levels ↓ IL-6
Sozmen et al. [111]	2016	BALB/c mice; AD-like model	100 μL of 0.5% DNFB	Resveratrol (30 mg/kg/day), administered repeatedly during the 6th week	↑ Epithelial thickness ↓ IL-25-, IL-33-, and TSLP-positive cells in the epithelium ↓ Caspase-3-positive cells in the epithelium
Bangash et al. [112]	2023	AD-like mouse model	200 μL of 1% DNCB dissolved in acetone–olive oil mixture (3:1) on the dorsal skin; 10 μL of 1% DNCB on the right ear; on the seventh day, the animals were resubjected to 0.5% DNCB on the dorsal skin (200 μL) and right ear (10 μL)	Daily topical pterostilbene at 0.2, 0.6, and 1% for 28 days	↓ IgE ↓ Epidermal thickness ↓ Oxidative stress markers in the skin ↓ IL-4 ↓ IL-6 ↓ TNF-α ↓ NF-κB
Kwack et al. [86]	2022	BALBc/AnNTac mice; AD-like model	100 μL of 2% DNCB	DNCB+PM10 +5 μM dieckol; DNCB+PM10 +5 μM punicalagin; DNCB + PM10 + 1 μM EGCG; DNCB + PM10 + 1 μM resveratrol; DNCB + PM10 + 10 μg/mL SHE	↓ IgE ↓ Spleen weight ↓ Epidermal thickness ↓ Mast cell counts ↓ IL-1β, IL-4, IL-6, IL-17α, IL-25, IL-31 ↓ TSLP Antioxidants prevented the downregulation of keratinocyte differentiation markers, including loricrin and fillagrin
Kjaer et al. [116]	2015	BALBc/AnNTac mice; Psoriasis-like model	Daily dose of 62.5 mg of 5% IMQ cream	400 mg/kg animal/day of resveratrol based on average food intake	↓ mRNA levels of IL-17A and IL-19
Shin et al. [120]	2020	NHEKs; Skin inflammation model	PM 0, 1.25 μg/mL, 3 μg/mL, 6 μg/mL, 12 μg/mL, 25 μg/mL, 50 μg/mL, 100 μg/mL, 200 μg/mL	Resveratrol 0, 0.01 μg/mL, 0.1 μg/mL, 1 μg/mL, 10 μg/mL, 50 μg/mL, 100 μM	↓ PM-induced aryl hydrocarbon receptor activation ↓ ROS formation in keratinocytes ↓ Subsequent cellular inflammatory response by inhibiting mitogen-activated protein kinase activation ↓ PM-induced cyclooxygenase-2/prostaglandin E2 and proinflammatory cytokine expression, including that of matrix metalloproteinase (MMP)-1, MMP-9, and interleukin-8
Moon et al. [121]	2020	HMC-1 cells; AD-like model	PMACI (0.128 ± 0.008 ng/mL)	Resveratrol (0.03, 0.3, and 3 μM)	↓ TSLP production and mRNA expression ↓ NF-κB activation ↓ IκBα phosphorylation ↓ Activation of receptor-interacting protein 2 and caspase-1 ↓ Upregulation of intracellular calcium
Lee et al. [122]	2016	HaCaT psoriasis-like model	Basal expression	Resveratrol (5, 10, 20, and 50 μM)	↑ apoptosis ↑ Sirt1 expression ↓ the p62 level ↓ Sirt1 Resveratrol-induced Sirt1 blocked Akt phosphorylation. ↓ Akt pathways by inducing Sirt1, thus leading to cell death

AD: atopic dermatitis; HDMs: house dust mites; HMGB1: high-mobility group box 1 protein; RAGE: receptors for advanced glycation end products; pNF-κB: phosphorylated nuclear factor-κB; p-PI3K: phosphoinositide 3-kinase; p-ERK1/2: phosphorylated extracellular signal-regulated kinase; IL-2Rα: interleukin-2 receptor α; IL-4: interleukin 4; IL-17α: interleukin 17 α; IL-31: interleukin 31; IL-25: interleukin 25; IL-33: interleukin 33; TSLP: thymic stromal lymphopoietin; IL-17A: interleukin 17A, IL-19: interleukin 19; NHEKs: normal human epidermal keratinocytes; HMC-1: human mast cell line; MMP-1: matrix metalloproteinase 1; MMP-9: matrix metalloproteinase 9; IκBα: NF-kappaB inhibitor alpha; RIP2: receptor-interacting protein-2 kinase; p62: protein p62; Sirt1: sirtulin 1, Akt: protein kinase B (PKB); HaCaT: human keratinocyte cell line; TEWL: transepidermal water loss; CXCL8: interleukin-8; 2,4-dinitrochlorobenzene (DNCB); PM: particulate matter; RR: resveratrol-enriched rice, PMACI: phorbol myristate acetate (PMA) plus calcium ionophore; IMQ: Imiquimod; EGCG: epigallocatechin gallate; SHE: Siegesbeckiae Herba extract.

## Data Availability

Not applicable.
